# Sternal resection and reconstruction for metastasis due to breast cancer: the Marlex sandwich technique and implantation of a pedicled latissimus dorsi musculocutaneous flap

**DOI:** 10.1186/s13019-019-0905-z

**Published:** 2019-04-18

**Authors:** Nozomu Motono, Kenichi Shimada, Toru Kamata, Hidetaka Uramoto

**Affiliations:** 1 Department of Thoracic Surgery, Kanazawa Medical University, 1-1 Daigaku, Uchinada, Ishikawa 920-0293 Japan; 2 Department of Plastic and Reconstructive Surgery, Kanazawa Medical University, 1-1 Daigaku, Uchinada, Ishikawa 920-0293 Japan; 3 Department of Breast Surgery, Keiju Medical Center, 64 Tomioka, Nanao, Ishikawa 926-8605 Japan

**Keywords:** Sternal resection, Metastasis, Breast cancer, Reconstruction

## Abstract

**Background:**

The treatment of hemotogenous solitary sternal metastases by breast cancer remains a controversial issue. Sternal resection for select patients might provide good long-term local control.

**Case presentation:**

A 63-year-old woman was admitted to our hospital with a mass at the sternum and right second to third costochondral cartilage. She had undergone bilateral mastectomy for breast cancer 13 years earlier. A percutaneous biopsy was performed, and the mass was diagnosed as solitary metastasis due to breast cancer. She received two courses of weekly paclitaxel and bevacizumab, and computed tomography (CT) revealed shrinking of the mass in the sternum. We performed surgical resection with curative intent for a multimodality approach. Parasternectomy and removal of the right second and third costochondral cartilage was performed. A prosthesis was created to fill the defect by sandwiching molded methylmethacrylate between polypropylene mesh. The prosthesis was fixed to the cut ends of the costochondral cartilage and the residual sternum. Finally, a harvested latissimus dorsi myoctaneous flap was transpositioned to cover the chest midline wound. Negative surgical margins at the stump of the sternum and costochondral cartilage were revealed.

**Conclusion:**

Parasternal resection and reconstruction by the Marlex sandwich technique and implantation of a pedicled latissimus dorsi myocutaneous flap for metastasis due to breast cancer was safely performed.

## Background

Sternal involvement of breast cancer can occur as a result of the hematological spread of tumor cells or direct spread from involved intra-mammary nodes [1]. Several authors recently reported good results for long-term palliation and an improved quality of life after sternectomy [2–5]. We performed parasternal resection and reconstruction via molded methylmethacrylate sandwiched between polypropylene mesh (Marlex sandwich technique) and an implanted pedicled latissimus dorsi myocutaneous flap for metastatic breast cancer.

## Case presentation

**Fig. 1:**
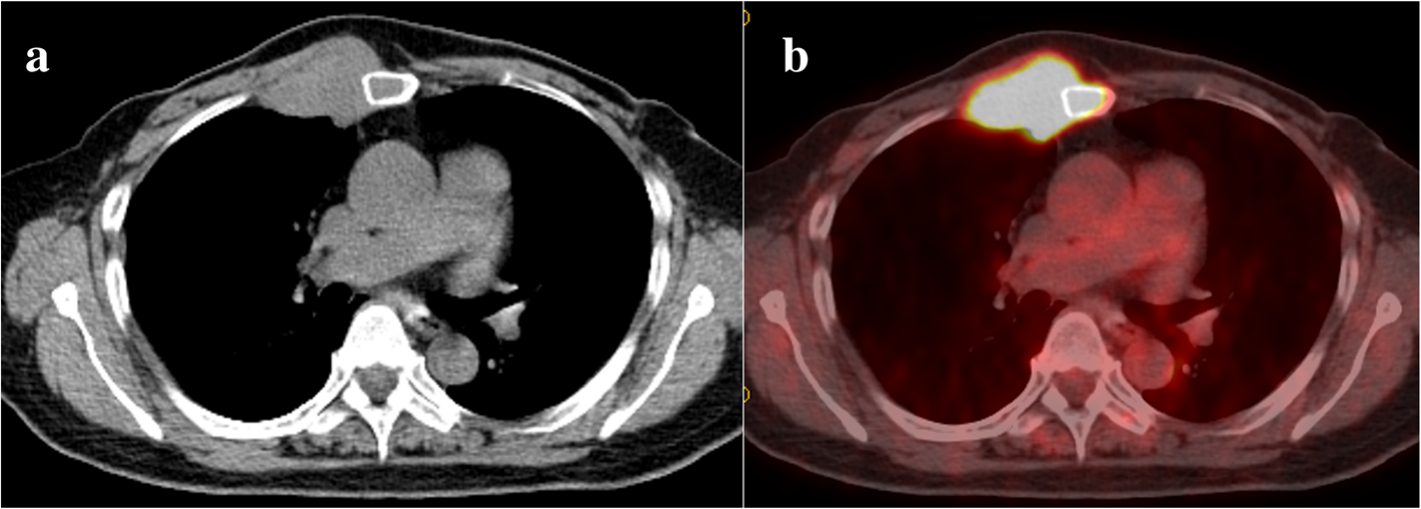
CT showing a 40 mm mass in sternum (**a**). PET-CT showing a standardized uptake value of [18f]-fluorodeoxyglucose of 7.30 on the mass in sternum (**b**)

**Fig. 2:**
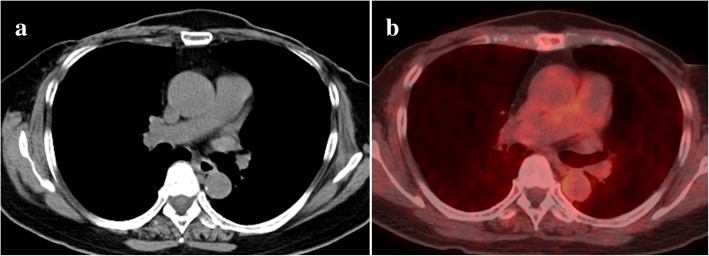
CT showing shrinking of the mass in sternum (**a**). The hot uptake on PET disappeared (**b**)

A 63-year-old woman admitted to our hospital with a mass at the sternum and right second to third costochondral cartilage. She had undergone bilateral mastectomy for breast cancer 13 years earlier. Computed tomography (CT) revealed a 40-mm mass in sternum (Fig. 1a). Positron emission tomography (PET) revealed the maximum of the standardized uptake value of [18f]-fluorodeoxyglucose to be 7.30 at the mass in the sternum, with no other lesions detected (Fig. 1b). A percutaneous biopsy was performed, and the mass was diagnosed as solitary metastasis due to breast cancer. She received two courses of weekly paclitaxel and bevacizumab, and CT revealed shrinking of the mass in sternum, while the hot uptake on PET disappeared (Fig. 2a and b). We performed surgical resection with curative intent for a multimodality approach.

**Fig. 3:**
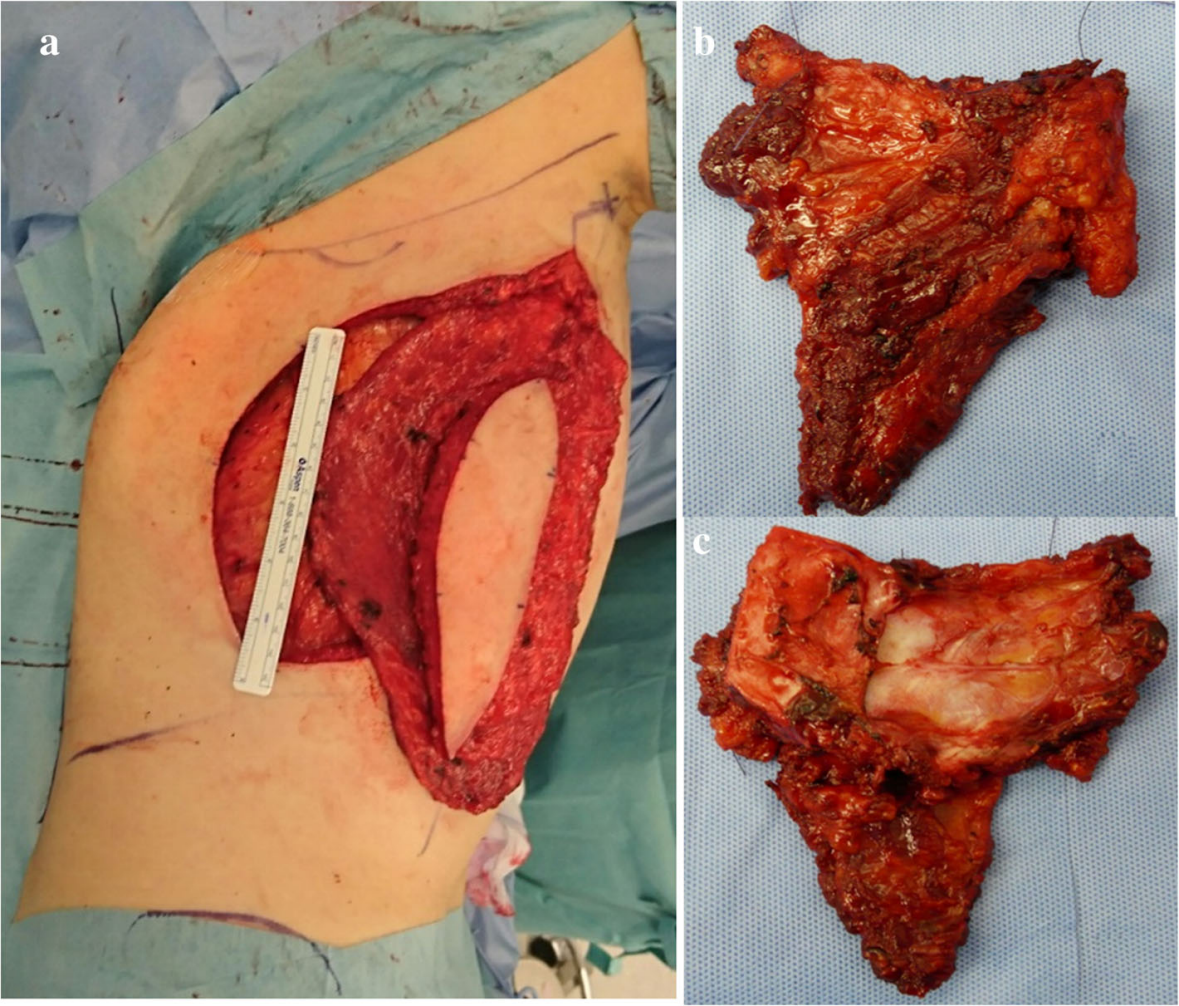
The latissimus dorsi myocutaneous flap was harvested with the patient in the left lateral position (**a**). Parasternectomy and removal of the right second and third costochondral cartilage was performed, as viewed from the front (**b**) and back (**c**)

**Fig. 4:**
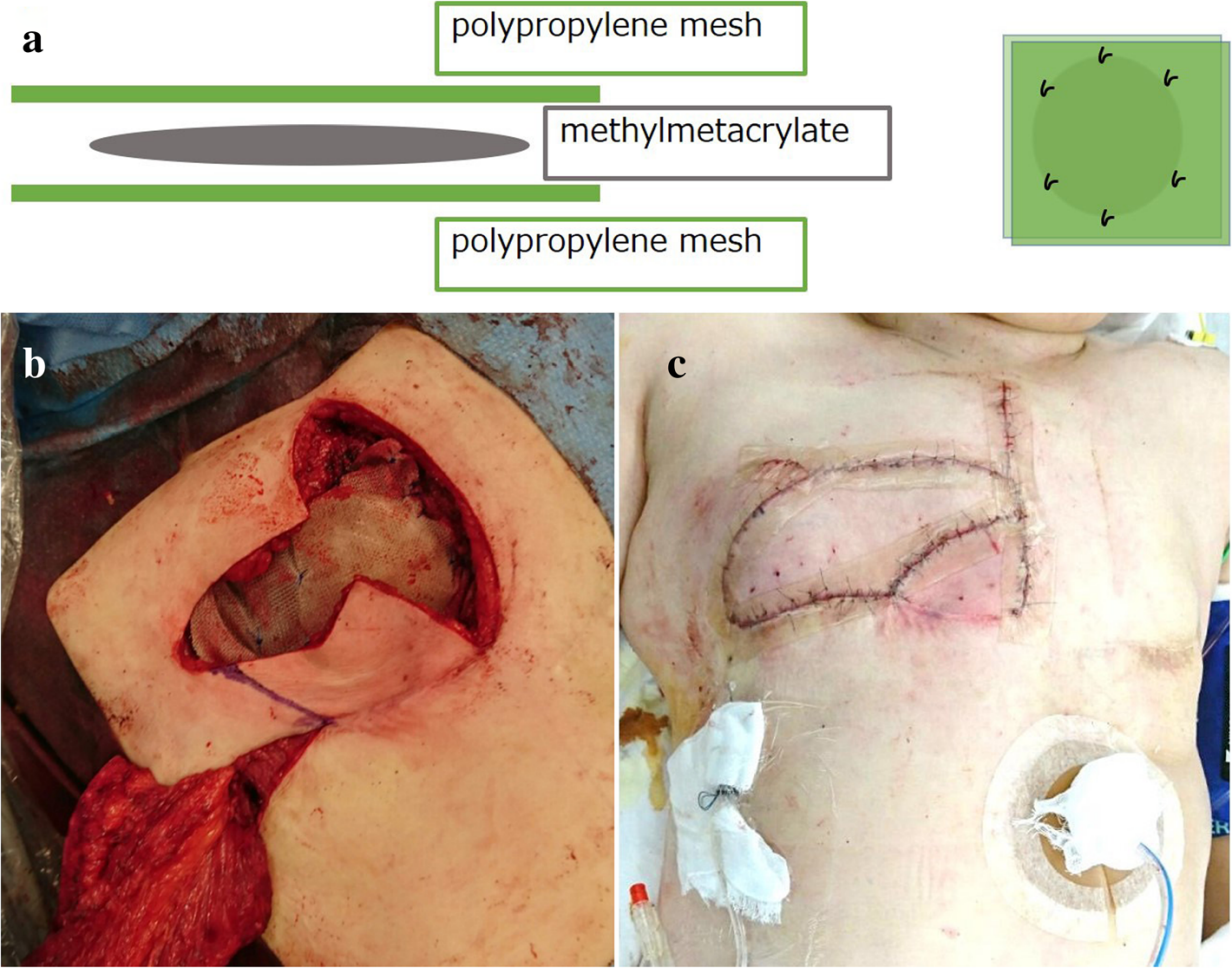
A prosthesis was created to fill the defect by sandwiching molded methylmethacrylate between polypropylene mesh (**a**). The prosthesis was fixed to the cut ends of the costochondral cartilage and the residual sternum (**b**). The harvested latissimus dorsi myoctaneous flap was transpositioned to cover the chest midline wound (**c**)

First, a latissimus dorsi myocutaneous flap was harvested with the patient in left lateral position (Fig. 3a). We could not assert that the tumor was not infiltrating the pectoralis major muscle and the subcutaneous layer. Therefore, we decided to remove these muscles. Parasternectomy and removal of the right second and third costochondral cartilage, the pectoralis major muscle, and the subcutaneous layer was performed in the supine position (Fig. 3b and c). A prosthesis was created to fill the defect by sandwiching molded methylmethacrylate between polypropylene mesh (Fig. 4a). The prosthesis was fixed to the cut ends of the costochondral cartilage and the residual sternum (Fig. 4b). Finally, the harvested latissimus dorsi myoctaneous flap was transpositioned to cover the chest midline wound and the prosthesis (Fig. 4c).

The postoperative course was uneventful, and her respiration was normal without paradoxical movement of the thorax or hypoxemia. A histological examination revealed that viable cells of metastatic breast cancer account for 30% of total cells, and cicatrization of metastatic breast cancer accounts for 70% of total cells in the sternum and the intercostal spaces. Immunohistochemistry revealed positivity for estrogen-receptor (ER) and progesterone-receptor (PR) and negativity for human epidermal growth factor receptor 2 (HER2). Furthermore, negative surgical margins at the stump of the sternum and costochondral cartilages were noted. Therefore, we decided not to do adjuvant therapy.

This patient has shown no complications and no recurrence in the four months since surgery. We planned to take CT every half year.

## Discussion and conclusions

Solitary sternal metastasis in patients with breast cancer is relatively uncommon, with reported incidences of 1.9–2.4% [6, 7]. The treatment of hemotogenous solitary sternal metastases by breast cancer remains a controversial issue. Sternal metastasis from breast cancer has been reported to remain localized in the sternum for a long time [2, 8]. This is thought to be due to the absence of a well-developed vascular network around the sternum. Therefore, sternal resection for select patients, such as those without mediastinal lymph node involvement, might provide good long-term local control [1, 2, 8]. Prognostic factors for the outcome of sternal resection were reported to be a disease-free survival of more than 24 months and axillary node-negative disease [9]. Another author reported that an interval lasting more than 10 years between primary treatment and chest wall resection was significantly correlated with a better overall and disease-free survival according to multivariate analysis [10].

However, patients who have triple-negative breast cancer with axillary node involvement have an increased likelihood of distant recurrence and of death compared with women with other types of cancer [11, 12]. Patients with triple-negative breast cancer experienced high rates of recurrence that peaked at three years after the diagnosis. In the case of our patient, sternectomy for metastasis due to breast cancer could be expected to have curative intent because of the > 10-year interval between primary treatment and sternectomy and her positive status for ER and PR.

Respiratory disturbance after resection of the anterior chest wall is a major problem, and different materials for chest wall reconstruction have been used [1–5]. The anatomic site and size of the skeletal defect should be taken into consideration when choosing the materials and methods for treatment (Table 1). Polypropylene mesh has been widely used because of its solidity, manageability, long-term tolerability, virtual absence of foreign body reactions or septic complications, and low cost. Although polypropylene mesh alone might be too weak for managing large chest wall defects, methylmethacrylate sandwiched between polypropylene mesh (Marlex sandwich technique) might offer the best results in terms of the fixation and protection of endothoracic organs [13, 14]. Furthermore, myocutaneous flaps have replaced other tissues, such as simple skin flaps or breast transposition, for the reconstruction of soft tissue defects, because of their safety and long-term stability [14]. In our case, the Marlex sandwich technique and pedicled latissimus dorsi myocutaneous flap implantation were performed for skeletal and soft tissue reconstruction, thereby preventing the paradoxical movement of the thorax and protecting the mediastinal organs from external trauma.

**Table 1:** Types and characteristics of prosthesis

	Polypropylene	Methylmetacrylate	Expanded- Polytetrafluoroethylene	Titanium
Tissue affinity	◎	△	◎	〇
Solidity	△	◎	〇	◎
Tolerability	△	◎	〇	◎
Infection resistance	◎	△	◎	〇
Manageability	◎	〇	◎	〇

We successfully performed parasternal resection and reconstruction by the Marlex sandwich technique with implantation of a pedicled latissimus dorsi myocutaneous flap for metastasis due to breast cancer. The anatomic site and size of the defect should be taken into consideration when choosing the materials and methods for treatment.

## Abbreviations

CT: Computed tomography
ER: Estrogen-receptor
HER2: Human epidermal growth factor receptor 2
PET: Positron emission tomography
PR: Progesteron-receptor

## References

[1] Noble J, Sirohi B, Ashley S, Ladas G, Smith I (2010). Sternal/parasternal resection for parasternal local recurrence in breast cancer. Breast.

[2] Nakamura H, Kawasaki N, Taguchi M, Tomoki K (2007). Reconstruction of the anterior chest wall after subtotal sternectomy for metastatic breast cancer: report of a case. Surg Today.

[3] Koppert LB, van Geel AN, Lans TE, van der Pol C, van Coevorden F, Wouters MWJM (2010). Sternal resection for sarcoma, recurrent breast cancer, and radiation-induced necrosis. Ann Thorac Surg.

[4] Veronesi G, Scanagatta P, Goldhirsch A, Rietjens M, Colleoni M, Pelosi G (2007). Results of chest wall resection for recurrent or locally advanced breast malignancies. Breast.

[5] Pameijer CR, Smith D, McCahill LE, Bimston DN, Wagman LD, Ellenforn JD (2005). Full thickness chest wall resection for recurrent breast carcinoma: an institutional review and meta-analysis. Am Surg.

[6] Kwai AH, Stomper PC, Kaplan WD (1988). Clinical significance of isolated scintigraphic sternal lesions in patients with breast cancer. J Nucl Med.

[7] Ohtake E, Murata H, Maruno H (1994). Bone scintigraphy in patients with breast cancer: malignant involvement of the sternum. Radiat Med.

[8] Noguchi S, Miyauchi K, Nishizawa Y, IMAOKA S, Koyama H, Iwanaga T (1988). Results of surgical treatment for sternal metastasis of breast cancer. Cancer.

[9] Chagpar AC, Meric-Bernstam F, Hunt KK, Ross MI, Cristofanilli M, Singletary SE (2003). Chest wall recurrence after mastectomy dose not always portend a dismal outcome. Ann Surg Oncol.

[10] van der Pol CC, van Geel AN, Menke-Pluymers MBE, Schmitz PIM, Lans TE (2009). Prognostic factors in 77 curative chest wall resections for isolated breast cancer recurrence. Ann Surg Oncol.

[11] Lee L, Keller A, Clemons M (2008). Sternal resection for recurrent breast cancer: a cautionary tale. Curr Oncol.

[12] Dent R, Trudeau M, Pritchard KI, Hanna WM, Kahn HK, Sawke CA (2007). Triple-negative breast cancer: clinical features and patterns of recurrence. Clin Cancer Res.

[13] Martini N, Huvos AG, Burt ME, Heelan RT, Bains MS, McCormack PM (1996). Predictors of survival in malignant tumors of sternum. J Thorac Cardiovasc Surg.

[14] Incarbone M, Nave M, Lequaglie C, Ravasi G, Pastorino U (1997). Sternal resection for primary or secondary tumors. J Thoracic Cardiovasc Surg.

